# Exploring the Impact of COVID-19 on Family Caregivers of Persons Living With Dementia in the Community

**DOI:** 10.1093/geroni/igab046.2112

**Published:** 2021-12-17

**Authors:** Carrie McAiney, Emma Conway, Melissa Koch, Laura Middleton, Heather Keller, Sherry Dupuis, Kate Dupuis, Jennifer Boger

**Affiliations:** 1 University of Waterloo, Waterloo, Ontario, Canada; 2 Schlegel-UW Research Institute for Aging, Waterloo, Ontario, Canada; 3 Waterloo University, Waterloo, Ontario, Canada

## Abstract

COVID-19 public health measures have significantly impacted persons living with dementia (PLWD) and family caregivers (FCGs). Given the restrictions on in-person services, many PLWD were not able to access their usual supports and activities, resulting in FCGs stepping in to support exercise, leisure, socialization, spirituality, and activities of daily living. At the same time, FCGs’ own support networks were significantly reduced or no longer available. We conducted in-depth qualitative interviews with 20 FCGs of PLWD in the community to explore the impact of COVID-19 on their well-being. Data were analyzed using thematic analysis. Caregiving during COVID-19 was described as ‘draining’ and ‘stressful’, with the support needs of PLWD increasing at a time when fewer supports were available. Reaching out to others, using technology, and setting boundaries were strategies FCGs used to cope. Despite the considerable impacts of COVID-19, FCGs of PLWD demonstrated their resilience in supporting themselves and their PLWD.

